# Recent updates in cancer immunotherapy: a comprehensive review and perspective of the 2018 China Cancer Immunotherapy Workshop in Beijing

**DOI:** 10.1186/s13045-018-0684-3

**Published:** 2018-12-21

**Authors:** Zihai Li, Wenru Song, Mark Rubinstein, Delong Liu

**Affiliations:** 1 Hollings Cancer Center, Medical University of South Carolina, Charleston, SC 29425 USA; 2 Chinese American Hematologist and Oncologist Network, New York, NY USA; 3 New York Medical College, New York, NY USA

## Abstract

The immune system is the hard-wired host defense mechanism against pathogens as well as cancer. Five years ago, we pondered the question if the era of cancer immunotherapy was upon us (Li et al., Exp Hem Oncol 2013). Exciting progresses have been made at all fronts since then, including (1) sweeping approval of six agents by the US Food and Drug Administration (FDA) to block the PD-1/PD-L1 pathway for treatment of 13 cancer types; (2) a paradigm shifting indication of PD-1 and CTLA4 blockers for the management of a broad class of cancers with DNA mismatch repair defect, the first-ever tissue agnostic approval of cancer drugs; (3) real world practice of adoptive T cell therapy with two CD19-directed chimeric antigen receptor T cell products (CAR-T) for relapsed and/or refractory B cell malignancies including acute lymphoid leukemia and diffuse large B cell lymphoma, signaling the birth of a field now known as synthetic immunology; (4) the award of 2018 Nobel Prize in Physiology and Medicine from the Nobel Committee to Tasuku Honjo and James Allison “for their discovery of cancer medicine by inhibition of negative immune regulation” (www.nobelprize.org/prizes/medicine/2018); and (5) the emerging new concept of normalizing rather than amplifying anti-tumor immunity for guiding the next wave of revolution in the field of immuno-oncology (IO) (Sanmamed and Chen, Cell 2018).

This article will highlight the significant developments of immune-oncology as of October 2018. The US FDA approved indications of all seven immune checkpoint blockers, and two CD19-directed CAR-T products are tabulated for easy references. We organized our discussion into the following sections: introduction, cell therapy, emerging immunotherapeutic strategies, expediting oncology drug development in an era of breakthrough therapies, new concepts in cancer immunology and immunotherapy, and concluding remarks. Many of these topics were covered by the 2018 China Cancer Immunotherapy Workshop in Beijing, the fourth annual conference co-organized by the Chinese American Hematologist and Oncologist Network (CAHON), China FDA (CFDA; now known as China National Medical Product Administration (NMPA)), and the Tsinghua University. We significantly expanded our discussion of important IO developments beyond what were covered in the conference, and proposed a new Three Rs conceptual framework for cancer immunotherapy, which is to reverse tolerance, rejuvenate the immune system, and restore immune homeostasis. We conclude that the future of immuno-oncology as a distinct discipline of cancer medicine has arrived.

## Introduction

It is estimated that by 2035, one quarter of the global populations will be directly affected by cancers (https://cancerprogressreport.org/Pages/cpr18-cancer-in-2018.aspx). There are five main therapeutic modalities for cancer: surgery, radiation, chemotherapy, targeted therapy, and immunotherapy. With a few exceptions, the first four modalities are focused squarely on cancer itself. Immunotherapy represents conceptually a unique way of dealing with cancer which is to focus on eliminating cancer indirectly by harnessing the power of the host’s immune system. The concept of cancer immunotherapy has been there for more than a century [1]. But it is only after the turn of this century that it has gained traction thanks to advancements in both basic immunology research [2] and the birth of immuno-oncology (IO) [3]. It is now established that as a genetically altered entity, cancer triggers both innate and adaptive immune response of the host during its evolution. Immune escape is recognized as one of the key hallmarks of cancer [4]. The implication of this fundamental and conceptual shift is significant because it inspires strategies to restore immunity to keep cancer permanently at bay, i.e., cure. Indeed, the discovery of both cellular and molecular mechanisms of cancer immune evasion fuels the development of IO agents, including immune checkpoint blockers against CTLA4, PD-1, and PD-L1 [5–7]. Importantly, the IO field is still at its early stage. There are more questions than answers. For example, less than one quarter of patients overall respond to PD-1/PD-L1 blockers. Frustratingly, there is a lack of biomarkers to predict who will respond and who will not to these agents. There has been no clear breakthrough to enhance efficacy of immune checkpoint inhibitors (ICIs). Furthermore, IO is shaking up the field of cancer medicine, but there is no clear and effective strategy to integrate immunotherapy into the conventional strategies for treating a majority of cancer types. Whereas ICIs have enjoyed unprecedented success, other immunotherapeutic strategies are not there yet in prime time. There are still no effective therapeutic vaccines. Approved cell therapy is also limited to B cell malignancies. The challenges IO field imposes to cancer medicine also include lack of adequate healthcare providers in this emerging field, and struggles of the regulatory agencies in crafting guidelines in steering and accelerating the clinical development of unconventional immune-regulatory agents. In light of these excitement and challenges, a much anticipated 2018 China Cancer Immunotherapy workshop was held in Beijing on June 30th and July 1st. This two-full-day meeting brought together IO experts from academia, industry, and government regulatory agencies around the world. This was the fourth time CAHON has partnered with the China FDA (joined also by Tsinghua University since 2017) to provide a high-level IO education conference annually to physicians, scientists, and drug developers in the industry to help advance IO in China and beyond.

### Clinical updates on checkpoint inhibitors

Two sessions of the conference were focused on clinical updates of ICIs. At the time of the conference (June 30–July 1, 2018), one CTLA4 blocker (Ipilumimab), two PD-1 inhibitors (Nivolumab and Pembrolizumab), and three PD-L1 antagonists (Durvalumab, Atezolizumab, and Avelumab) were approved by the US FDA for various indications (Table 1). Subsequently, the third PD-1 blocker Cemiplimab was approved for the treatment of patients with metastatic cutaneous squamous cell carcinoma (CSCC) or locally advanced CSCC who are not candidates for curative surgery or curative radiation. This is based on encouraging clinical study including the positive study by Migden et al. who performed an expansion phase I study as well as the pivotal phase 2 study for patients with metastatic disease CSCC [8]. Patients received cemiplimab i.v. at 3 mg/kg of body weight every 2 weeks and were assessed for clinical response every 8 weeks. Deep response in the phase 1 expansion cohort of patients was observed in 50% of patients (*n* = 26), which was reproduced in the phase 2 study, with response rate in 28 of 59 patients (47%; 95% CI, 34 to 61). This response appeared to be durable, exceeding 6 months in most patients without observed new immune-related adverse events (irAEs).

**Table 1:**
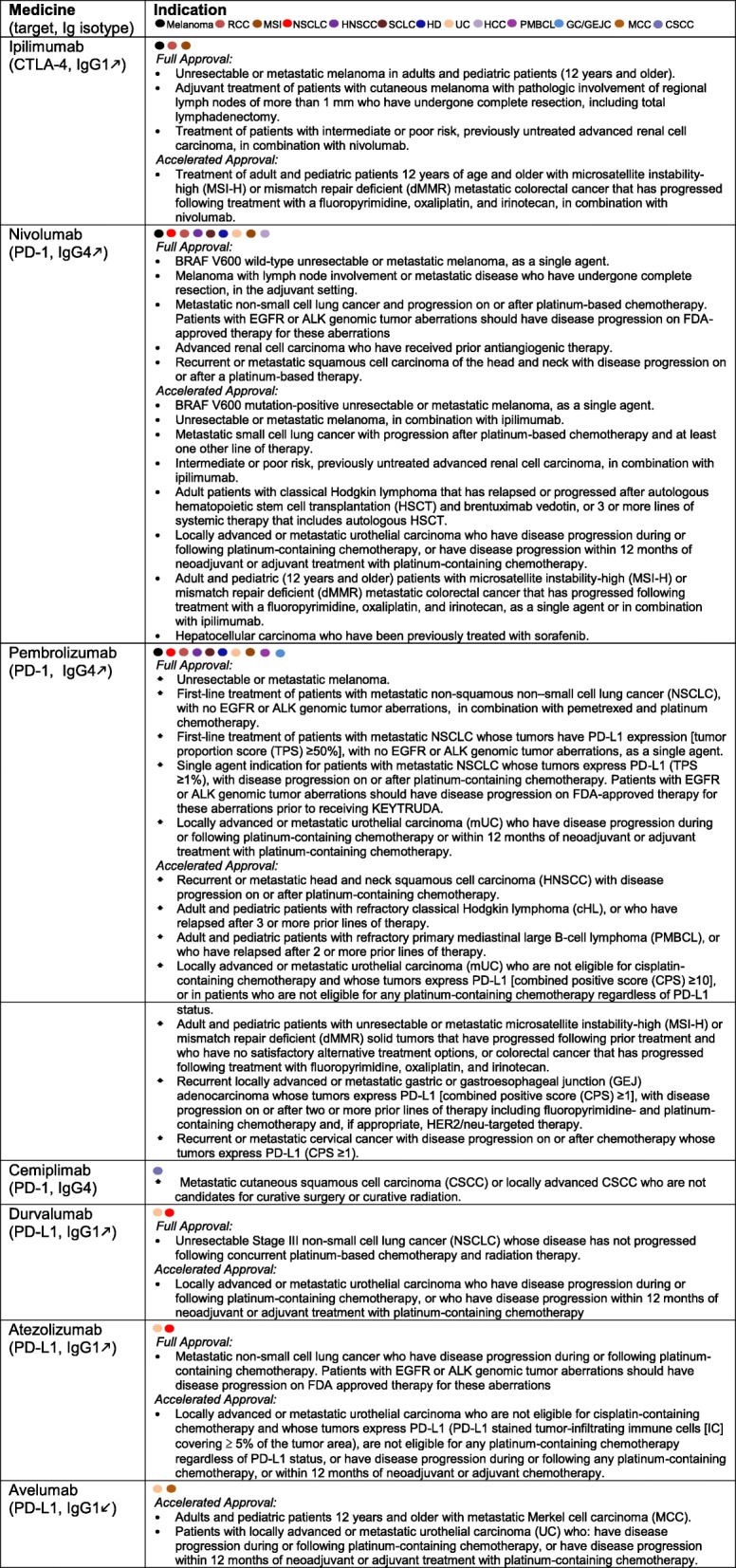
US FDA approved immune checkpoint blockers for cancer immunotherapy as of Oct 2018

Altogether at the time of writing this report (Oct 27, 2018), seven ICIs have been approved collectively for the standard treatment of a total of 13 cancer types. Excitingly, the US FDA has also granted accelerated approval for Nivolumab (with and without Ipilumimab) and Pembrolizumab for the management of advanced cancers with DNA mismatch repair deficiency, regardless of the histology of the cancer types, marking for the first time the approval of cancer medicine in a tissue-agnostic fashion. The clinical experiences with these agents were highlighted in designated talks by Weijing Sun (gastrointestinal cancer, University of Kansas), Yiping Yang (hematological malignancy, Duke University), Jun Zhu (lymphoma, Beijing University Cancer Hospital), Mario Sznol (melanoma, Yale University), Jun Guo (melanoma, Beijing University Cancer Hospital), Yilong Wu (lung cancer, Guangdong General Hospital), Shukui Qin (hepatocellular carcinoma, Nanjing PLA Hospital), and Jingshong Zhang (genitourinary cancer, Lee Moffitt Cancer Center). In addition to the agents approved in the USA, researchers from China also presented exciting data regarding PD-1 inhibitors and other IO agents developed in China, by the following companies: Hengrui, Innovent, Beigene, Jun Shi, 3DMed, Zai Lab, and I-Mab.

Of note, clear differences do exist in both the distribution and biology of cancers between the West and the East, underscoring the importance of conducting IO trials in China rather than totally depending on clinical experience in other parts of the world, for guiding the IO approval process. In Asia, liver and upper gastrointestinal cancers are epidemics which may have different underlying biology. Whereas both acral and mucosal melanoma are exceedingly rare in the USA at 5% and 1–2% of all melanomas, Jun Guo pointed out that in China these two subsets could be 49.4% and 22.6% respectively [9]. Sznol highlighted the experience with stage IV melanoma with ICIs. Nivolumab plus Ipilimumab is an approved strategy in this setting. Among all the patients treated with this combination (*N* = 94) in the initial phase I trial at a follow-up of 30.3 to 55.0 months, the 3-year overall survival rate was 63% and median overall survival had not been reached at the time of the publication of the analysis [10]. The investigators reported 42% objective response rate by modified WHO criteria, and median duration of response was 22.3 months. Unfortunately, the improved efficacy is also accompanied by the increased incidence of severe (grade 3 and 4) treatment-related adverse events at 59%. Nonetheless, the 3-year OS rate of 63% in advanced melanoma highlighted the significant clinical utility and efficacy of ICIs. Interestingly, the appearance of CD21^low^ B cells in the peripheral blood in a study with a small cohort of patients appears to predict immune-related adverse events (irAEs) without affecting efficacy [11]. Sznol also outlined practical principle in the management of irAEs, by recommending the following: (a) ruling out the possibility of disease progression or infection, (b) following established guidelines [12–14], (c) having low threshold to start corticosteroids and admit the patients to the hospital for inpatient care, (d) maintaining high dose steroids for at least 1 week and tapering slowly over 30–40 days, and (e) discontinuing IO agents permanently for grade IV irAEs. These points were further underscored by Helen Chen (US National Cancer Institute), who cautioned of risks of enhanced toxicities with immunotherapy combinations with targeted agents. Several interesting combinations have since been discontinued due to increased toxicities, including durvalumab plus osimertinib (pneumonitis), tremelimumab plus suninitinib (renal failure), crizotinib plus nivolumab (hepatic toxicities) [15], and nivolumab plus pazopanib (hepatic toxicities).

### Cell therapy

2018 marked the year when IO enjoys unprecedented growth at many fronts. In a comprehensive analysis of the global IO landscape, Tang and colleagues found that in the span of just 1 year (September 2017 to September 2018), there was a 67% increase in the number of active agents in the global IO pipeline (2031 versus 3394) [16]. Impressively, the cell therapy class had the largest growth—a whopping 113% increase in the number of active agents. While it may be argued that bone marrow or hematopoietic stem cell transplantation represents the best-established cell therapy for human malignancy, CD19-targeted CAR-T cells for B cell neoplasms open up the imagination of scientists in the field in perhaps signaling what more could come in this extraordinary space. There are two approved CD19-CAR-T cell platforms: Tisagenlecleucel (Kymriah) and Axicabtagene ciloleucel (Yescarta), which have similarities and differences (Table 2). Both agents are autologous peripheral T cells engineered ex vivo to express a transmembrane chimeric antigen receptor composed of an extracellular antigen-specific single chain antibody and an intracellular T cell signaling domain. Both agents utilize single chain anti-CD19 antibody to target B cells, and CD3ζ intracellular signaling motif to deliver primary activating signals to T cells. However, tisagenlecleucel employs additional CD137 (4-1BB) signaling for co-stimulation as opposed to axicabtagene which does so with a CD28 signaling cassette. Both agents have been approved in the USA for the treatment of relapsed or refractory large B cell lymphoma after two or more lines of systemic therapy. Tisagenlecleucel is additionally approved for the treatment of patients up to 25 years of age with B cell precursor acute lymphoblastic leukemia (ALL) that is refractory or in second or later relapse.

**Table 2:** Comparison of two US FDA approved CAR-T products for B cell malignancies

Medicine	Signaling motifs	Dosage	Indication
YESCARTA Axicabtagene ciloleucel (Yescarta)	CD28 and CD3ζ	2 × 10^6^ CAR-positive viable T cells per kg body weight, with a maximum of 2 × 10^8^ CAR-positive viable T cells.	• Adult patients with relapsed or refractory large B cell lymphoma after two or more lines of systemic therapy, including diffuse large B cell lymphoma (DLBCL) not otherwise specified, primary mediastinal large B cell lymphoma, high grade B cell lymphoma, and DLBCL arising from follicular lymphoma.
KYMRIAH™ Tisagenlecleucel (Kymriah)	CD137 (4-1BB) and CD3ζ	Pediatric and young adult B cell ALL (up to 25 years of age): • For patients 50 kg or less, administer 0.2 to 5.0 × 10^6^ CAR-positive viable T cells per kg body weight intravenously. • For patients above 50 kg, administer 0.1 to 2.5 × 10^8^ total CAR-positive viable T cells (non-weight based) intravenously. Adult relapsed or refractory diffuse large B cell lymphoma: • Administer 0.6 to 6.0 × 10^8^ CAR-positive viable T cells intravenously.	• Patients up to 25 years of age with B cell precursor acute lymphoblastic leukemia (ALL) that is refractory or in second or later relapse. • Adult patients with relapsed or refractory (r/r) large B cell lymphoma after two or more lines of systemic therapy including diffuse large B cell lymphoma (DLBCL) not otherwise specified, high grade B cell lymphoma and DLBCL arising from follicular lymphoma, excluding primary central nervous system lymphoma.

The presentation by Patrick Hwu (MD Anderson Cancer Center), Ke Liu (US FDA), Weidong Han (Army Hospital in Beijing), Sen Zhuang (Johnson & Johnson), and Chunyan Gao (China National Medical Product Administration) discussed a number of important issues about cell therapy, as follows:

#### Flavors of cell therapy

Cellular products in the clinical application and testing including hematopoietic stem cells, CAR-T cells against CD19, and other targets, T cells engineered to express T cell receptor with known specificity (TCR-T), tumor-reactive or tumor-infiltrating T cells isolated and expanded from cancer patients (otherwise known as endogenous T cells, or ETC), polyclonal tumor-reactive T cells (tumor-infiltrating T cells, or TILs) isolated from the tumor, NK cells, NKT cells, dendritic cells, etc. Patrick Hwu summarized the MDACC experience in their TIL therapy program for 74 metastatic melanoma patients from 2007 to 2017 [17]. They found that the best overall response for the entire cohort was 42%: 47% in 43 ICIs-naïve patients, 38% when patients were exposed to anti-CTLA4 alone (21 patients) and 33% if also exposed to anti-PD1 (9 patients) prior to TIL therapy. Median overall survival was 17.3 months; 24.6 months in CTLA4-naïve patients and 8.6 months in patients with prior CTLA4 blockade. The latter patients were infused with fewer TILs and experienced a shorter duration of response. They found that infusion of higher numbers of TIL with CD8 predominance and expression of BTLA (B And T Lymphocyte Associated) by the tumor cells correlated with improved response in anti-CTLA4 naïve patients, but not in anti-CTLA4 refractory patients. Baseline serum levels of IL9 predicted response to TIL therapy, while curiously TIL persistence, tumor recognition, and mutation burden did not correlate with outcome. They concluded that there are deleterious effects of prior exposure to anti-CTLA4 on TIL therapy response. Hwu discussed a number of strategies to improve TIL cell therapy based on rational thinking and preclinical data including stably expressing dominant negative TGFβ receptor II in the TIL products to overcome immune suppression in the tumor microenvironment [18] and transduction of T cells with CXCR2 to allow them to better migrate to the tumor sites [19]. Importantly, recent breakthroughs in genomic medicine and informatics enable the detection of neoantigen epitopes and subsequent expansion of antigen-specific TILs using these antigens in the context of appropriate HLA. Adoptive transfer with neoantigen-specific T cells has been shown to mediate objective clinical responses in patients with metastatic bile duct, colon, and cervical cancers, as well as triple negative breast cancers [20–23]. The practical challenge of this approach is similar to what CD19-CAR-T technology faced almost 10 years ago [24], which is to determine how to move exciting proof-of-principle science from the academic settings to real world clinical practice.

#### Targeting antigens of CAR-T cells

Without doubt, the bottle neck to prevent CAR-T technology to be widely used clinically is the lack of optimal target antigens for a majority of cancers like CD19 for B cell malignancies. Patients with B cell aplasia can live relatively healthy with maintenance therapy of intravenous immunoglobulins from normal donors. In comparison, life cannot be sustained with lack of myeloid cells which is why CAR-T based strategy has not found significant success for the treatment of myelodysplastic syndrome or acute myelogenous leukemia. To circumvent this problem, Kim et al. deleted CD33 from the normal human hematopoietic stem cells and transplanted into rhesus macaques with long-term multilineage engraftment with normal myeloid function [25]. These CD33-deficient cells then allow CD33-targeted CAR-T therapy for efficient elimination of CD33^+^ leukemia without myelotoxicity. For plasma cell disorder, it is a different story. One can afford the ablation of normal plasma cells in order to eradicate malignant plasma clone with CAR-T based strategy. In this regard, BCMA (B cell maturation antigen)-CAR-T-based strategy, LCAR-B38M, was discussed by Sen Zhuang (Johnson and Johnson). A confirmation clinical trial has started in the USA, followed by the original encouraging data in China with 35 patients who participated in the study. In that study, all patients responded to the therapy, with 94% showing sustained complete or near-complete remission [26, 27]. As of July 2018, a total of 74 patients have been treated with LCAR-B38M, updated by Frank Fan (Legend Biotech). In various phases of clinical trials are also CAR-T cells targeting other cell surface antigens including GD2, HER2, CD20, EBV antigen, mesothelin, CD33, CD22, CD30, CD123, EGFR, PSMA, WT1, GPC3, CD38, EGFRvIII, MUC1, PDL1, and neoantigens [28].

#### Dual, switchable, off-the-shelf, SUPRA CAR-T, etc.

Weidong Han discussed multiple efforts in designing safer and more effective CAR-T strategies [29]. By gene editing methodology, genes encoding human leukocyte antigen (HLA) molecules and endogenous T cell receptors (TCRs) can be deleted and these T cells will then be transduced to express CAR-T construct, followed by expansion in vitro, cryopreservation, and aliquoting. These products can then be used for any patients whose cancer express the target of the CAR-T cells. This effort is ongoing for CD19^+^ B cell malignancies. One switchable CAR-T cell strategy is to make these T cells to bind to a specific peptide that is genetically engrafted onto a tumor-binding Fab molecule. The “switch” acts as a bridge between target and effector cells, which can be titrated due to the relatively short half-life of the Fab fragment. It was found that such a strategy worked well in a preclinical model against human Her2^+^ cancer in a mouse xenograft system [30]. Multiple other strategies have been developed to control CAR-T activity including using combinatorial antigen-sensing system [31], or engineering a built-in suicide system in the CAR to allow physicians to switch off CAR-T when unwanted toxicities emerge. Another exciting strategy was the so-called SUPRA CAR, which is a split, universal, and programmable system [32]. It has a two-component receptor system composed of a universal receptor (zipCAR) expressed on T cells and a tumor-targeting scFv adaptor (zipFv). Both the receptor and scFv adaptor contains leucine zipper, allowing targeting of multiple antigens without further genetic manipulations of a patient’s T cells. This strategy had remarkable successes in preclinical models against several types of cancer by simultaneously targeting multiple antigens using one batch of engineered zipCAR-T cells.

#### Regulatory challenges

Ke Liu (US FDA) and Chenyan Gao (CFDA) discussed the regulatory challenges imposed by the intense interests of the public in CAR-T technology. Like other products, the regulatory agencies uphold three basic principles when it comes to evaluate cell therapy products for approval: substantial evidence of efficacy, acceptable safety, and appropriate patient population. Ke Liu cautioned that both CD19-CAR-T products on the market carry black box warning for cytokine release syndrome and neurotoxicity. He emphasized that much work needs to be done in solid tumor space with focus on target identification, understanding and enhancing CAR-T cell tracking and homing to tumor site, to maximize the clinical benefit.

### Emerging immunotherapeutic strategies

A number of exciting progresses have been made to usher the field of IO into the next phase, which is beyond ICIs against PD-1, PD-L1, and CTLA4. Space is limited to cover all of the new developments. What were highlighted in 2018 China Cancer Immunotherapy Workshop included the following:

#### Search for other surface-bound immune checkpoint molecules

Mounting evidence suggest there are additional immune checkpoint molecules to constrain tumor-reactive T cells. Through single-cell RNAseq and proteomics approach, a recent work from Anderson Regev, Kuchroo and colleagues discovered a module of co-inhibitory receptors in both CD4^+^ and CD8^+^ T cells that includes PD-1, TIM-3, LAG-3, TIGIT, activated protein C receptor (PROCR), and podoplanin (PDPN) [33]. The module of co-inhibitory receptors is shared by non-responsive T cells in several physiological contexts and is driven by the immunoregulatory cytokine IL-27. Importantly, they found that PRDM1 and c-MAF serve as cooperative transcription regulators of the co-inhibitory module. Chen Dong (Tsinghua University) updated his work on B7 superfamily member 1 (B7S1), also called B7-H4, B7x, or VTCN1. They found that the increased B7S1 expression on myeloid cells from patients with hepatocellular carcinoma correlated with CD8^+^ T cell dysfunction [34]. The receptor of B7S1, yet to be defined, is co-expressed with PD-1 but not Tim-3 on T cells during activation, which promotes T cell exhaustion. Intriguingly, blocking of both B7S1 and PD-1 synergistically enhanced anti-tumor immune responses. Using a high throughput functional screening strategy, the team of Lieping Chen (Yale) discovered a cell surface molecule that is expressed by a subset of myeloid cells and tumor cells (ovarian, lung, bladder, pancreas, head, and neck cancer) called Siglec15 (unpublished). Although the receptor for Siglec15 on T cells has not been molecularly defined yet, Siglec clearly plays negative roles for T cell activation and function by inducing suppressive myeloid cells. In an unprecedented pace, NC318, a Siglec15 targeting antibody, has already entered a phase 1/2 clinical trial in patients with advanced or metastatic solid tumors.

#### Immunogenomics and precision immunotherapy

Precision immunotherapy requires understanding of both tumor microenvironment (the tumor) and macroenvironment (the host, i.e., the patient). A comprehensive presentation was delivered by Elizabeth Jaffee (Johns Hopkins), Tim Chan (Memorial Sloan-Kettering), Drew Pardoll (Johns Hopkins), and Siwen Hu-Lieskovan (UCLA). Immunogenomics is a rapid expanding area that allows researchers to interrogate and understand how changes of the cancer genome affect immunity or treatment responsiveness. For example, understanding tumor mutation burden (TMB), immunoediting score etc. will enable researchers and physicians to guide ICI therapy [35, 36]. Understanding TCR repertoire, neoantigen epitopes and HLA haplotypes will facilitate effort in neoantigen vaccine development and cell therapy. Jaffee discussed their meta-analysis results of patients on anti-PD-1/PD-L1 agents whose exome sequencing information were available [37]. They found a strong relationship between the tumor mutational burden and the activity of anti–PD-1 therapies across multiple cancer types. Their analysis allowed them to calculate objective response rate (ORR) with a linear correlation formula: ORR = 10.8 × log_e_(*X*) − 0.7, where “*X*” is the number of coding somatic mutations per megabase of DNA. Validation of this finding with future prospective trials shall be helpful to guide the selection of patients for ICIs. Catherine Wu and her colleagues have identified a subcluster of MAGE-A cancer-germline antigens, located within a narrow 75 kb region of chromosome Xq28, that predicts resistance uniquely to blockade of CTLA4, but not PD-1 [38]. Tim Chan discussed the exciting study from his group that highlighted the importance of mutation of specific genes correlating to ICI responsiveness. They reported that somatic mutations in SERPINB3 and SERPINB4 are associated with survival after anti-CTLA4 immunotherapy in two independent cohorts of patients with melanoma (*n* = 174), although the underlying mechanism is unclear [39]. Furthermore, Tim Chan’s group determined the HLA class I genotype of 1535 advanced cancer patients treated with ICIs. They found that maximal heterozygosity at HLA class loci correlated with improved overall survival compared with patients who were homozygous for at least one HLA locus. Curiously, in two independent melanoma cohorts, patients with the HLA-B44 had extended survival, whereas the HLA-B62 supertype (including HLA-B*15:01) or somatic loss of heterozygosity at HLA class I was associated with poor outcome [40]. Hu-Lieskovan discussed several lines of work in UCLA, including a remarkable 70% clinical response of patients with desmoplastic melanoma to PD-1 blockers, which correlated with high tumor mutation burden and frequent NF1 mutations in this unique subset of melanoma patients [41]. PD-1 blocker-based therapy ultimately depends on CD8^+^ T cells and IFNγ for cancer eradication. Not surprisingly, loss of function mutations of MHC class I (e.g., loss of β2m) and key IFNγ signaling molecules JAK1/2 in the cancer are associated with intrinsic resistance to anti-PD-1 therapy [42, 43]. Perhaps, a more striking example of impact of cancer genomics on ICI treatment is the status of microsatellite instability-high (MSI-H) or DNA mismatch repair deficiency (dMMR) in the tumors [44–47]. About ~ 50% patients with advanced cancers and the defect in the mismatch repair pathway will derive clinical benefit in response to nivolumab or pembrolizumab. Genomics study of cancer can also shed light on the mechanism of immune evasion. For example, a multi-omic analysis of 1211 colorectal cancer primary tumors reveals that it should be possible to better monitor resistance in the 15% of cases that respond to ICI therapy and also to use WNT signaling inhibitors to reverse immune exclusion in the 85% of cases that currently do not [48]. Genomic and immunologic studies have also uncovered specific driver mutations correlated with lower (CTNNB1, NRAS, or IDH1) or higher (BRAF, TP53, or CASP8) leukocyte levels across all cancers [49]. The oncogenic pathways [50], such as PTEN loss [51, 52], and activation of the WNT/β-catenin signaling pathway [53] have been shown to lead to poor T cell infiltration and function in the tumor microenvironment.

In the field of personal neoantigen vaccines [54], there have been several high profile proof-of-principle studies. Ott et al. demonstrated the feasibility, safety, and immunogenicity of a neoantigen vaccine platform (up to 20 personized HLA-A/B-restricted peptides plus poly-ICLC as adjuvant) that targets advanced melanoma [55]. Evidence for T cells discriminating mutated from wild-type antigens was shown for some patients. Another group tested RNA-based poly-neo-epitope approach for patients with melanoma [56]. They found evidence suggesting that patients developed T cell responses against multiple vaccine neo-epitopes and increased T cell infiltration and neo-epitope-specific killing of autologous tumor cells in post-vaccination resected metastases. Although the sample size is too low to conclude the clinical utility for all of these studies, the neoantigen-based approach may prove to be useful in the adjuvant setting, particularly in combination with ICIs. Pardoll discussed their allele-integrated deep learning framework for improving class I and class II HLA-binding predictions, which may be useful for future neoantigen vaccine effort and also the expansion of tumor antigen-specific T cells [57]. Jaffee also discussed the Hopkins experience on the combination of neoantigen vaccine and ICIs and other IO agents such as CD40 agonist, CXCR4 inhibitor, and agents that target CD47, CSF1R, IDO, TGF-β, A2A, etc. But these studies are mostly at the preclinical stage. Undoubtedly, effective cancer immunotherapy depends on robust priming of tumor-specific T cells, enabling T cells to infiltrate the tumors and ensuring effective mechanism to prevent T cell dysfunction due to hostile tumor microenvironment.

#### Targeting soluble immune checkpoint

Besides cell surface immune checkpoint molecules, there are multiple soluble immune suppressive factors that play important roles in maintaining immune homeostasis. These factors include but, are not limited to, prostaglandins, nitric oxide, IL-10, TGF-β, IL-33, IL-35, IL-4, IL-13, IL-37, and VEGF. Thorsson et al. performed an extensive immunogenomic analysis of more than 10,000 tumors comprising 33 diverse cancer types by mining the TCGA data [49]. They identified six immune subtypes, including wound healing, IFNγ dominant, inflammatory, lymphocyte depleted, immunologically quiet, and TGF-β dominant. The importance of TGF-β in driving immune suppression and its place in targeted cancer immunotherapy was discussed by Zihai Li (Medical University of South Carolina). Accumulating evidence suggest that TGF-β is a key mechanism for resistance to blockade to PD-1/PD-L1 in multiple cancer types including bladder cancer [58], colorectal cancer [59], and others. However, TGF-β targeting alone, either with small molecule inhibitors of the signaling pathway or anti-TGF-β antibody, has met with limited clinical success due to narrow therapeutic window and heterogeneity of cancer biology in patient populations [60]. Recently, a bifunctional molecule targeting both PD-L1 and TGF-β, called M7824, has been developed [61]. M7824 is a chimeric molecule containing the N-terminal region of fully human IgG1 against human PD-L1 and the C-terminal TGF-β neutralizing trap component from the extracellular domain of the human TGF-β receptor 2. Preclinically, M7824 efficiently binds PD-L1 and TGF-β in vivo and suppressed tumor growth and metastasis more effectively than treatment with either an anti-PD-L1 antibody or TGF-β trap alone in syngeneic mouse models. Encouragingly, M7824 treatment resulted in activation of both the innate and adaptive immune systems, and synergize with radiotherapy or chemotherapy in mouse models. Gulley and his colleagues conducted a phase I open-label trial of M7824 in 19 heavily pretreated patients with advanced solid tumors [62]. They found that M7824 hit and saturated the targets at > 1 mg/kg. Clinical efficacy was seen across all dose levels, including one ongoing confirmed complete response (cervical cancer), two durable confirmed partial responses (PR; pancreatic cancer, anal cancer), one near-PR (cervical cancer), and two cases of prolonged stable disease at study entry (pancreatic cancer, carcinoid). Ongoing clinical studies of M7824 include treatment of patients with colorectal cancer, HPV^+^ malignancies, and a planned trial to compare M7824 with pembrolizumab as a first-line treatment in patients with PD-L1-expressing advanced non-small cell lung cancer (NSCLC).

Another development in the TGF-β field is the discovery of a cell surface dock receptor for activation of latent TGF-β, called Glycoprotein A Repetitions Predominant (GARP) [63]. Encoded by *LRRC32*, GARP has its restricted expression by regulatory T cells [64, 65] and platelets [66] in normal individuals. Whether GARP is expressed by cancer cells and how it impacts cancer have been investigated. It was found that GARP promotes oncogenesis and immune tolerance by enriching and activating latent TGF-β in the tumor microenvironment [67]. GARP expression and folding depends on a pro-oncogenic molecular chaperone gp96 in the endoplasmic reticulum [68]. Importantly, by both gain- and loss-of-function studies using normal mammary gland epithelial cells and carcinomas, GARP was found to increase the bioactivity of TGF-β and promote malignant transformation in immune-deficient mice [67]. In immune-intact mice, over-expression of GARP in mammary carcinomas drives expansion of regulatory T cells, which contributes to enhanced cancer progression and metastasis [67]. Intriguingly, Rachidi et al. discovered that constitutive expression of GARP on platelets is the most important mechanism of TGF-β activation in vivo, placing platelets squarely in the immune suppressive workforce [69]. Finally, several GARP-specific monoclonal antibodies have been reported. In one case, GARP-targeted antibody was shown to reduce regulatory T cell function in vivo [70]. In another case, a competitive anti-GARP antibody to block the binding between GARP and LTGF-β showed significant activity to perturb metastasis in an orthotopic breast cancer model [67]. Thus, a gp96-GARP-TGF-β switch is a novel oncogenic mechanism that can be exploited for both diagnostic and therapeutic purposes.

#### Rational combination therapy

The success of ICIs against the broad spectrum of cancers has now reset the baseline of IO. The focus of the IO field for the last 5 years has not been on replacing ICIs but on how to improve their efficacy for a greater proportion of patients. This topic became the central theme of the conference and was touched upon by almost all the speakers especially Lei Zheng (Johns Hopkins), Yang-Xin Fu (UT Southwestern), and Elizabeth Jaffee (Johns Hopkins). There are existing approved combination therapies with nivolumab and ipilimumab for treatment of advanced melanoma, renal cell carcinoma, MSI high tumors, etc. (Table 1). The first-line treatment of patients with metastatic NSCLC, without EGFR or ALK genomic tumor aberrations, is also in combination with pemetrexed and platinum chemotherapy. Not surprisingly, there has been an impressive increase in new combination studies in the past 5 years. Analyses of the Cancer Research Institute database by Tang and his colleagues show that in 2017 alone, 469 new studies were started, with a target enrollment of 52,539 patients, principally being combined with anti-PD-1/L1 agents [71]. For example, a phase 1b clinical trial was conducted to study the impact of oncolytic virotherapy with talimogene laherparepvec in combination with pembrolizumab for advanced melanoma [72]. Confirmed objective response rate was 62%, with a complete response rate of 33% per immune-related response criteria. Responders had increased CD8^+^ T cells, elevated PD-L1 protein expression, as well as IFN-γ gene expression on several cell subsets in the tumors [72]. Excitingly, during the 2018 European Society for Medical Oncology (ESMO) annual meeting, a positive result of Phase 3 KEYNOTE-426 trial was announced by the study sponsors. This study tests pembrolizumab plus axitinib versus sunitinib alone in treatment-naive advanced/metastatic renal cell carcinoma (mRCC) (NCT02853331). A total of 861 patients with advanced or metastatic RCC were randomized to receive frontline treatment with pembrolizumab (200 mg IV every 3 weeks) plus axitinib (5 mg orally twice daily) for up to 24 months, or sunitinib (50 mg orally once daily for 4 weeks followed by no treatment for 2 weeks, continuously). No new safety concerns were raised. Although the final data is not available yet, the earlier study leading to the trial indeed offered encouraging results to potentially change the standard of practice for the treatment of advanced RCC [73]. There are also interests in combining cytokine-directed therapy with ICIs, as in the case of M7824 mentioned above to block TGFβ and PD-L1 simultaneously. The roles of common γ-chain cytokines including second generation IL-2 and IL-15 in boosting ICIs have also gained attention. For example, one encouraging phase Ib study has shown the utility of the combination of nivolumab and ALT-803 for patients with metastatic NSCLC [74]. ALT-803 is a homo-dimer of IL-15Rα-Fc (IgG1) bound with recombinant IL-15^N72D^ [75]. A pegylated IL-2, NKTR-214, which is a pro-drug and has the preferential release of the active IL-2 in the tumor microenvironment, has an excellent preclinical activity [76] and is now being tested in combination with ICIs for multiple malignancies in multiple settings. However, abundant evidence also sends a cautionary note to the field that the effective combination therapy is easy said than done. Indoleamine 2,3-dioxygenase 1 (IDO1) is a rate-limiting enzyme in the tryptophan catabolism and plays important roles in immune suppression [77]. It makes rational sense to combine inhibitors of PD-1 and IDO for cancer immunotherapy. However, despite the encouraging early phase data [78, 79], a recent phase III ECHO 301 trial testing the combination of epacadostat (an orally bioavailable IDO inhibitor) with pembrolizumab in melanoma did not show superior outcome compared to pembrolizumab alone [80].

Lei Zheng (Johns Hopkins) discussed rational thought process in designing combination therapy. Ideally, the two combined agents or modalities shall have single agent efficacy (such as PD-1 and CTLA4 inhibitors), non-overlapping mechanism of actions and toxicities (e.g., ICIs and cytotoxic agents), and being used for the right populations of patients selected carefully based on precision biomarkers. The last point is important for IO agents because, for example, one would not want to treat T cell excluded tumors with agents that reverse T cell exhaustion only [81]. In patients when the frequency of tumor-reactive T cells is low, strategies need to be brought forward with vaccinations (proper antigens with new generation of adjuvants), adoptive transfer of tumor-reactive T cells, and mechanisms to amplify T cell responses with co-stimulatory agents (such as CD137 agonist), survival cytokines, and means to tame immune tolerance mechanisms such as turning off regulatory T cells.

Yang-Xin Fu (UT Southwestern) discussed several novel agents and their application preclinically by targeting both innate and adaptive immunity, which highlighted a number of important principles for developing future IP agents. LIGHT (TNFSF14) is immune stimulatory cytokine. A bifunctional molecule has been generated to link anti-EGFR antibody on the one arm with a three tandem LIGHT fused with Fc domain on the other arm. This α-EGFR-LIGHT fusion protein was shown to be able to overcome resistance to anti-PD-1 and convert non-T cell infiltrating (“cold”) tumor to tumors with increased infiltrating T cells (“hot”) tumor. Interestingly, a series of works from Fu and his colleagues showed that therapeutic roles of commonly used antibodies in oncology (against Her2, EGFR and CD20 for example) are dependent on T cells [82–84], providing a rationale for combining these antibodies with ICIs for cancer immunotherapy. Another intriguing strategy is targeting CD47, a “do not-eat-me” signal on macrophages and other antigen-presenting cells for cancer immunotherapy [85, 86]. A humanized anti-CD47 antibody, Hu5F9-G4, has demonstrated therapeutic efficacy in vitro and in vivo in patient-derived orthotopic xenograft models on five aggressive pediatric brain tumors [87]. The roles of CD47-targeting monotherapy might be problematic due to the significant side effect of causing red blood cell destruction and lack of preference of targeting tumor-infiltrating macrophages. However, by priming (1 mg/kg) and maintenance (10–30 mg/kg weekly starting week 2) dosing, the anemia induced by Hu5F9-G4 can be mitigated. When it was combined with rituximab, promising activity was seen in patients with refractory B cell lymphoma in a phase 1b study involving 22 patients [88]. Liu et al. recently revealed that CD47 and PD-L1 on tumor cells coordinately suppress innate and adaptive sensing to evade immune control. Targeted blockade of both CD47 and PD-L1 on tumor cells with a bispecific anti-PD-L1-SIRPα agent showed significantly enhanced tumor targeting and therapeutic efficacy comparing with monotherapy [89]. This finding makes sense because the cancer therapeutic effect of targeting CD47 also depends on CD8^+^ T cells [90].

### Expediting oncology drug development in an era of breakthrough therapies

Richard Pazdur (US FDA) provided unique perspectives on oncology drug development including in the area of IO. The FDA oversees medical and food industries that are a quarter of the America’s expenditures. It is responsible for assurance of the safety, efficacy, and security of these products. The hematology and oncology division has established disease-specific structure that is akin to current academic models, including Division of Oncology Products 1 (dealing with genitourinary, breast, and gynecologic cancer), Division of Oncology Products 2 (thoracic, head and neck, gastrointestinal, melanoma-sarcoma, pediatric/neuroendocrine/rare tumors), Division of Hematology Products (benign hematology products, lymphoma, leukemias, and transplant), and Division of Hematology and Oncology Toxicology (toxicologists supporting each division). Oncology drug development and approval are unique comparing with other therapeutic areas in that cancer deals with severe and life-threatening diseases, it has a large public interest which needs to expedite drugs, the area has different risk tolerance for side effects, there are strong active advocacy groups, the area enjoys one of the most active biomedical research, 50% of breakthrough therapies are in oncology space, and the oncology drug approval often utilizes biomarkers for subgroup patient selection. Regarding efficacy endpoints, FDA has moved away from overall response rate and transitioned to more emphasis on overall survival which means putting more weight on how patients “feel, function, or survive.” The explosion of IO field also coincides with the introduction of FDA expedited programs, leading from fast track to breakthrough therapy, to priority review, and eventually to accelerated approval. All ICIs now have indications based on the accelerated approval mechanism which requires post-marketing clinical trials to be underway at the time of approval. FDA also welcomes novel seamless trial design in drug development as opposed to the traditional rigid three discrete phases of clinical trials (I, II, and III). This is especially important for IO drug such as CD19-CAR-T which cannot be ethically tested in the phase III trial setting against relapsed and refractory B cell leukemia and lymphoma because the standard care offers negligible hope for controlling these diseases. Yangmin Ning (US FDA) discussed in details the nuts and bolts of the “Breakthrough Therapy Designation” program which started in 2012. It is designed to accelerate the approval of life-saving drugs with confirmed evidence that likely changes the standard of care for patients. There are two requirements for designating Breakthrough Therapy: (a) life-threatening diseases with unmet medical needs and (b) preliminary clinical evidence showing substantial improvement over available or existing therapies. It is important to keep in mind that such a designation does not mean an approval for marketing and implication of cure and is not restricted to oncology.

Zhimin Yang (China’s NMPA) discussed the oncology drug approval approach in China which mirrors the practice in the USA. Leading up to June 25, 2018, there were 193 trials with PD-1 blockers in China that were listed in Clinicaltrials.gov. She discussed a number of issues that are not new but made more prominent in the approval consideration for IO medicine: patient selection (cancer types, histology, biomarkers, upfront vs salvage therapy, etc.), efficacy, monotherapy vs combination therapy, and manageable toxicity. Regarding clinical trial design, for cancer types or stages that do not have a standard care option, NMPA also allows single-arm trial to gain regulatory approval. Undoubtedly, the future of regulation of IO development will be more dependent on bio-marker selection of patients, rather than histological types of diseases. It will also be based on mechanistic insights of the medicine rather than empiric reasoning. All of these considerations will hopefully lead to the launch of much more effective and less toxic IO medicine into the clinics.

### New concept in cancer immunology and immunotherapy

**Fig. 1:**
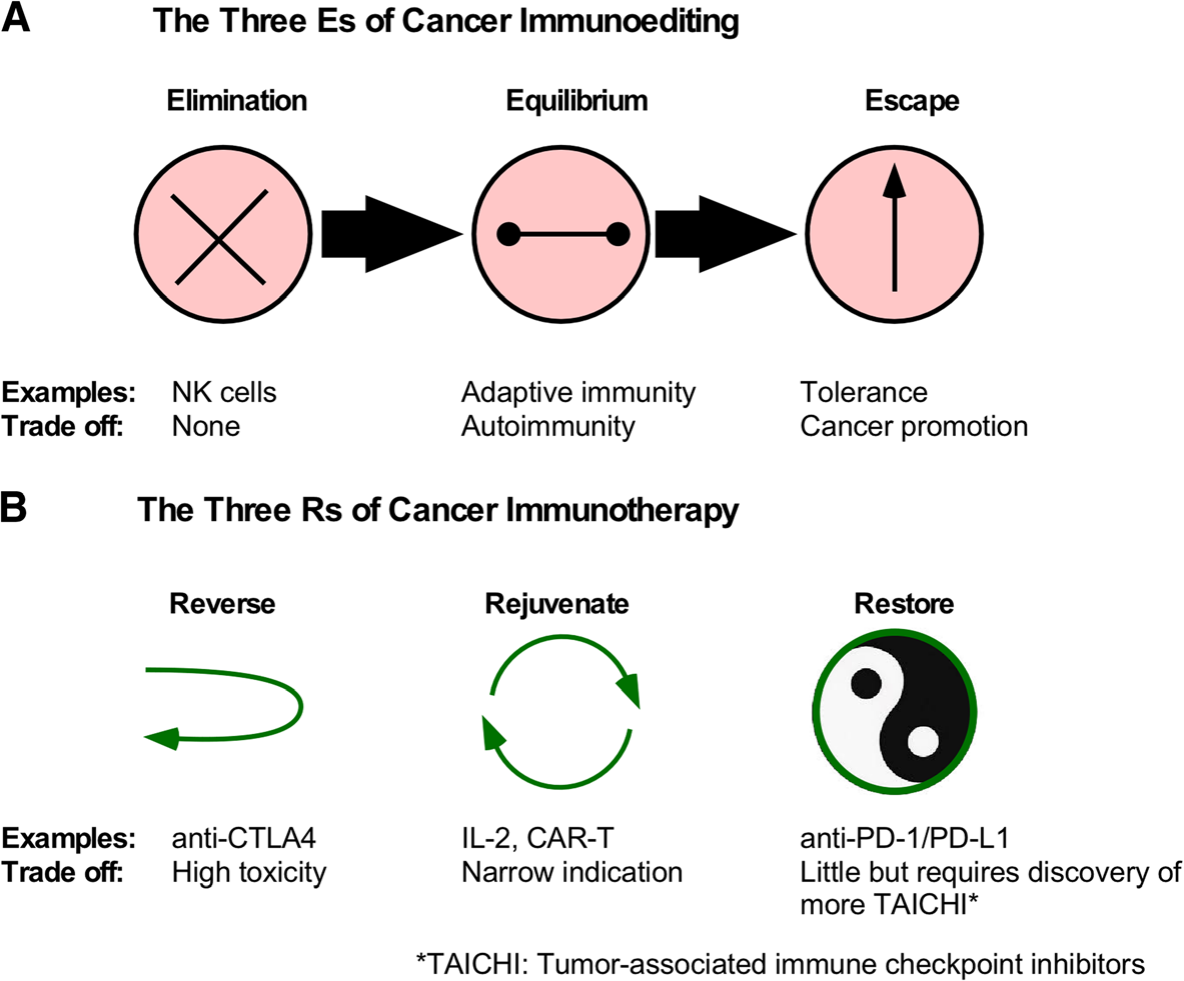
Principles of immunoediting and immunotherapy of cancer. **a** The 3Es model of cancer immunoediting is schematically shown, along with examples of the immune response and the trade-offs in each phase. **b** The 3Rs model of cancer immunotherapy divides treatment modalities into three distinct mode of actions: to reverse, rejuvenate, and restore anti-cancer immunity

Cancer immunotherapy has come a long way. It has been fueled by the basic understanding of the immune system and the unveiling of the dynamic interaction between the host immunity and the transformed cells during oncogenesis. Experimental data coupled with human epidemiology studies have established that during the ontogeny of cancer, immune response against cancer undergoes three functional phases, namely elimination of the cancer cells, equilibrium between cancer and the host immunity, and escape of cancers from the immunological attack [2] (Fig. 1a). This three Es model is helpful for guiding the development of immunotherapeutic strategies which deal primarily with cancer immune escape. Accordingly, in principle, cancer immunotherapy can be summarized with a framework of three Rs, which are to reverse tolerance, rejuvenate the immune system, and restore the immune homeostasis (Fig. 1b). Each of the modalities has its unique characteristics with the first 2 Rs associated with significant toxicities and a limited scope of application at present. The Holy Grail of cancer immunotherapy is the third R, as argued and championed by Lieping Chen (Yale University) to be the process of normalizing the immune response (i.e., dial back the immune editing to the elimination phase). This idea, presented at the conference and further elaborated elegantly in a recent publication by Sanmamed and Chen [3], emphasizes the concept of normalization of anti-tumor immunity in the tumor microenvironment that has aberrant expression of tumor-associated immune regulatory molecules. We would like to coin the term TAICHI for tumor-associated immune checkpoint inhibitory molecules to describe these molecular entities. PD-L1 is a prime example of TAICHI. It is important to point out that the Three R strategies may need to be deployed at the same time, or given sequentially in order to maximize the chance of cancer cure.

In an attempt to discover more TAICHI for cancer immunotherapy, Lieping Chen and his colleagues performed functional screening for cell surface molecules that inhibit T cell activation. As discussed above, they found that Siglec-15 has previously unknown immunosuppressive roles through promoting the survival and differentiation of suppressive myeloid cells and negatively regulates T cell function. Anti-Siglec antibody has already entered the clinical trial for the dose-defining study in patients with advanced solid tumors.

ICIs, CAR-T cells, etc., are currently used primarily in patients with advanced cancers. Drew Pardoll (Johns Hopkins) argued that the maximal benefit of these agents has not been realized. Immunotherapy of early stage of cancers before intervention by conventional strategies might induce the best benefit and shall be considered. This concept is supported by encouraging results with ICIs used in the neoadjuvant settings, for the treatment of cancers such as melanoma [91], bladder cancer [92], and head and neck cancer [93].

To determine what anti-PD-1 agents do to the tumor microenvironment in early-stage diseases, Forde et al. tested the roles of nivolumab in the neoadjuvant setting for adults with untreated, surgically resectable early (stages I, II, or IIIA) NSCLC. Nivolumab was associated with few side effects, did not delay surgery, and induced a major pathological response in 45% of resected tumors. As predicted, the tumor mutational burden correlates with the pathological response to PD-1 blockade, and the treatment induced expansion of neoantigen-specific T-cell clones in peripheral blood [94]. Future studies will need to address if upfront immunotherapy can change the natural history of the diseases and if so what will be the roles (or lack of) of surgery if pathological complete responses can be accomplished. Conceptually, studies like this will push IO experts and the regulatory agencies to move IO medicine much earlier in the management of cancer rather than using it as the last reserve of treatment.

Weiping Zou (University of Michigan) discussed the holistic approach in cancer immunotherapy, by examining not only tumor microenvironment for genomic alterations and changes of immune infiltration pattern, but also looking at the macroenvironment of the patients, including metabolism, microbiome, and other co-morbidities. Regarding PD-1/L-1-based immunotherapy, work from Arlene Sharpe, Weiping Zou, Yang-Xin Fu, and others showed that PD-L1 expression on tumor-associated professional antigen-presenting cells, as opposed tumor cells, could be the major target for anti-PD-1/L1 responsiveness for some cancers [95–97]. Zou and his colleagues also asked a provocative question regarding the roles of the immune system in chemoresistance. They found that CD8^+^ T cells, via the JAK/STAT1 pathway, can abrogate fibroblast-mediated chemoresistance in ovarian cancer model through upregulation of gamma-glutamyltransferases and repression of system xc(−) cystine and glutamate antiporter [98]. In colon cancer, it was found that *Fusobacterium (F.) nucleatum* in the gut was able to promote colorectal cancer resistance to chemotherapy by activating innate immunity and the autophagy pathway and thereby altering colorectal cancer chemotherapeutic response. Thus, how chemotherapy and microbiome contribute to reducing cancer burden and death shall also be re-examined in the era of IO. Finally, a case was made to effectively target regulatory T cells (Tregs) as a major path forward for immunotherapy [99]. Multiple agents have been tested for depleting Tregs or inactivating Treg function, including antibodies that block CTLA-4, GITR, GARP, CCR4, CD25, PD-1, OX-40, and LAG3, and small molecule inhibitors against PI3Kδ, PTEN, IDO, EZH2, and ZAP70 [99, 100]. However, a cautionary note was provided that even apoptotic Tregs can release high levels of ATP, which are then converted to adenosine via CD39 and CD73 to suppress T cell immunity [101].

Finally, the unique and distinct role of non-profit organizations in promoting and supporting IO development was shared by representatives from American Associaton of Cancer Research (AACR) (Elizabeth Jaffee), Society of Immunotherapy of Cancer (SITC) (Mario Sznol), Cancer Drug Development Forum (CDDF) (Heinz Zwierzina), Parker Institute for Cancer Immunotherapy (PICI) (Ramy Ibrahim), Chinese Society of Clinical Oncology (CSCO) (Jin Li), and National Foundation for Cancer Research (NFCR) (Sajuan Ba).

### Conclusive remarks

Without a doubt, the era of immuno-oncology is upon us. The true significance of IO medicine in the battle of mankind against cancer may still not be fully appreciated until a decade or so later. The broad activity of PD-1/PD-L1 agents against cancer has cemented the notion that immune escape is indeed a fundamental hallmark of cancer. Such a revelation raises hope and lifts the cloud of years’ frustration and failure over the field of cancer immunology. Thus, the Nobel Committee is right to acknowledge that the work of blocking inhibitory signals for treatment of cancer is Nobel-worthy. It is the long-term and painstaking basic research in immunology that has made this feat possible. James Allison has relentlessly pursued anti-CTLA4 antibody for cancer immunotherapy [102, 103] and has been the champion leading the current IO revolution. Studying basic mechanism of activation-induced cell death of lymphocytes, Tasuko Honjo cloned PD-1 [104] and showed later the importance of PD-1 pathway in negatively regulating T cell function [105–107]. However, the list of mavericks and pioneers of IO who have contributed to the Nobel-worthy work is long, including Lieping Chen who first cloned PD-L1 (also known as B7-H1) [108] and showed its inhibitory function [109, 110], IFNγ-inducibility [109], and its roles in constraining T cell immunity against cancer [109]; Gordon Freeman who collaborated with Honjo to establish the receptor-ligand interaction between PD-1 and PD-L1 [106]; Pierre Goldstein who first cloned CTLA4 [111]; and Jeffrey Bluestone [112, 113], Tak Mak [114], and Arlene Sharpe [113] who demonstrated the inhibitory function of CTLA4. Thanks to the work of those and others, the stage of IO has been set and the script has been written. We are here for a remarkable thriller which we hope will put smiles on face of all of our patients.

## Abbreviations

AACR: American Association for Cancer Research
BCMA: B cell maturation antigen
CAHON: Chinese American Hematologist and Oncologist Network
CAR-T: T cells expressing chimeric antigen receptor
CDDF: Cancer Drug Development Forum
CFDA: China Food and Drug Administration
CSCC: Cutaneous squamous cell carcinoma
CSCO: Chinese Society of Clinical Oncology
CTLA-4: Cytotoxic T-Lymphocyte Associated Protein 4
dMMR: DNA mismatch repair deficiency
ETC: Endogenous T cells
GARP: Glycoprotein A repetitions predominant
HLA: Human leukocyte antigen
ICI: Immune checkpoint inhibitor
IDO: Indoleamine 2,3-dioxygenase
NFCR: National Foundation for Cancer Research
NMPA: China National Medical Product Administration
NSCLC: Non-small cell lung cancer
PD-1: Programmed cell death protein 1
PD-L1: Ligand 1 from programmed cell death protein 1
SITC: Society of Immunotherapy of Cancer
TAICHI: Tumor-associated immune checkpoint inhibitory molecule
US FDA: The United States Food and Drug Administration
